# Related Factors of Suicidal Ideation among North Korean Refugee Youth in South Korea

**DOI:** 10.3390/ijerph15081694

**Published:** 2018-08-09

**Authors:** Subin Park, Soo Jung Rim, Jin Yong Jun

**Affiliations:** 1 Department of Research Planning, Mental Health Research Institute, National Center for Mental Health, Seoul 04933, Korea; subin-21@hanmail.net (S.P.); soojung0411@gmail.com (S.J.R.); 2 Department of Psychiatry, National Center for Mental Health, Seoul 04933, Korea

**Keywords:** refugee, adolescent, risk factor, protective factor

## Abstract

This study investigated the factors associated with suicidal ideation among 174 North Korean refugees (aged 13–27 years) residing in South Korea. Specifically, we compared sociodemographic, familial, social, and psychological characteristics between participants with and without suicidal ideation. Twenty-nine refugees (16.7%) had exhibited suicidal ideation in the past 12 months. These refugees had significantly lower levels of familial cohesion (*U* = 1459.0; *p* < 0.001), self-esteem (*U* = 1032.0; *p* < 0.001), and resilience (*U* = 1190.0; *p* < 0.001), as well as higher levels of expressional suppression (*U* = 1202.5; *p* < 0.001) and post-traumatic stress disorder symptoms (*U* = 1303.0; *p* = 0.001), (with Cohen’s *d* > 0.5), compared to those without suicidal ideation. A multiple logistic regression analysis showed that the level of emotional suppression and familial cohesion were significantly associated with suicidal ideation, after controlling for the other variables. Familial and individual interventions, particularly those focused on encouraging emotional expression and familial cohesion, will be useful for North Korean refugee youth, who have a high risk of suicide.

## 1. Introduction

Almost half of the individuals who emigrate for reasons such as armed conflict, persecution, and economic pressure in their home countries are children and adolescents [1]. These children and youth not only suffer during their escape or displacement from their home country, but also after their arrival and during settlement in their new country [2]. Accordingly, researchers have been focusing greater attention on how to support their development following this rapid transition.

The number of North Korean refugees (NKRs) that settled in South Korea exceeded 30,000 in 2016, and around 40% of these were individuals aged 10–29 years [3]. Like other refugees, NKRs are exposed to traumatic events not only when residing in North Korea, but also during their escape [4]. Even after they have settled in South Korea, NKRs often struggle to adapt to their new culture [5]. NKR youths, in particular, face obstacles such as gaps in physical health, compared to same-age peers, perceived discrimination, culture shock, and low social support, all of which can lead to the high drop-out rates from school and unemployment after settling in South Korea [6,7]. Correspondingly, NKR youths often suffer from anxiety, depression, and post-traumatic stress disorder (PTSD) [8,9,10], all of which are associated with suicide ideation and attempts [11,12].

In 2009, suicide was reported as the leading cause of death among youth (i.e., 15–19 years old) in South Korea [13,14]. Since NKR youths are exposed to traumatic events and suffer in adapting to their new environment, they naturally require more clinical attention [15]; in fact, NKR youths appear to have a much higher prevalence of suicidal ideation compared to South Korean youths [16]. Since suicidal ideation is considered to have a strong association with suicidal behavior [17], it is important to investigate the factors related to suicidal ideation to prevent suicide. Klonsky and May [18], proposed the Three Step Theory of Suicide. The first step states that for an individual to have suicidal ideation, pain and feeling of hopelessness are needed. Then, if an individual has a suicidal ideation and also feels unconnected to life, he or she will move on to the next step: strong ideation. Finally, if an individual is capable of making a suicide attempt, he or she will proceed to the final step, actual attempt. According to this theory, suicide happens in a stepwise manner, so finding factors that are related to suicidal ideation is important to prevent individuals from proceeding to the next step. Since NKR youths experience traumatic events, the factors that are related to suicidal ideation of this group could be different from the general public. Therefore, exploring which factors are associated with this vulnerable group’s suicidal ideation is important. However, to our knowledge, there has been no study investigating the factors influencing the suicidal ideation of this vulnerable group.

This study investigated the factors associated with suicidal ideation among NKR youths to prevent them from proceeding to the next step of the Three Step Theory. When exploring factors related to suicidal ideation of NKR youths, we have referred to a conceptual framework used by Reed and colleagues [1], which is based on the ecological model by Bronfenbrenner [19]. This conceptual framework [1] is used to explain the risk and protective factors for mental health of refugees. In this framework, the protective and risk factors of mental health are divided into four levels: individual (e.g., physical, psychological, or developmental disorders, age, sex, and exposure to violence), family (e.g., family composition, bereavement, and functioning), community (community social support), and societal (for example, language or cultural differences). We concentrated on factors from individual, family, and community level that could influence NKR youth’s mental health. Factors included in the societal level were not included in the present study, due to aspects of data availability. Specifically, we explored sociodemographic (individual level), psychological (individual level), familial (family level), and social (community level) factors that might influence suicidal ideation of NKR youths in South Korea.

## 2. Materials and Methods

### 2.1. Participants and Procedure

We recruited NKR youths between 2017 and 2018 from two alternative schools for NKRs who are preparing for qualification examinations for middle- or high-school graduation. NKR youths who settle in South Korea go through a mental health screening program, of which this study was a part. All individuals who participated in the program were selected for recruitment, and consequently, a total of 174 NKR youths aged 13–27 years participated in this study. Participants completed a self-report questionnaire on their sociodemographic, familial, social, and psychological characteristics, as well as whether they had ever seriously considered suicide in the last 12 months. Informed consent was obtained from all participants. Among 174 participants, 29 (16.7%) answered that they had seriously thought about suicide in the past 12 months. The study was reviewed and approved by the institutional review board of the National Center for Mental Health (No. 116271-2017-11).

### 2.2. Measurements

#### 2.2.1. Sociodemographic Characteristics

Information on sociodemographic characteristics (i.e., age, sex, birthplace, parental educational levels, and type of residence), which is part of the individual level, was obtained.

#### 2.2.2. Familial and Social Support

Familial (family level) and social support (community level) were assessed using the FACES III questionnaire [20,21], which comprises 20 items. There are two major parameters of family functioning that FACES III explores: cohesion and adaptability. Cohesion is assessed via statements such as “Family members know each other’s close friends” and “Our family does things together”. Adaptability is assessed via items such as “When problems arise we compromise” and “Family members say what they want”. Each item is rated on a scale ranging from 1 (“almost never”) to 5 (“almost always”).

We also assessed participants’ level of psychological support from others by asking participants “How much psychological support do you currently receive from your family, relatives, friends, and others around you?” Practical support was assessed by asking participants “How much practical support do you currently receive from your family, relatives, friends, and others around you?” For both questions, the responses were given on a 10-point Likert scale (1 = “not at all”; 10 = “receive enough support”).

#### 2.2.3. Psychological Characteristics

Psychological characteristics (i.e., resilience, self-esteem, cognitive style, impulsivity, and PTSD symptoms), which are included in the individual level, were assessed. We utilized the Brief Resilience Scale [22] to measure resilience (defined as the self-perceived ability to bounce back from stress). This scale contains three positive items (i.e., items 1, 3, and 5) and three negative items (i.e., items 2, 4, and 6). Each item is rated on a five-point scale (1 = “strongly disagree”; 5 = “strongly agree”). Negative items were reverse-scored. The total score ranges from 6 to 30, with higher scores indicating higher resilience.

The Rosenberg Self-Esteem Scale [23] was utilized to measure global self-worth. This scale contains ten items, and each rated on a 5-point Likert scale (1 = “strongly disagree” to 5 = “strongly agree”). The score ranges from 10 to 50, with higher scores indicating higher self-esteem.

The Emotion Regulation Questionnaire (ERQ) [24,25] was utilized to assess cognitive reappraisal (e.g., “When I’m faced with a stressful situation, I make myself think about it in a way that helps me stay calm”) and expressive suppression (e.g., “I control my emotions by not expressing them”), which are two emotion regulation strategies. Each item was rated on a 7-point Likert scale (1 = “strongly disagree”; 7 = “strongly agree”). Higher scores on each subscale indicate the respondent’s greater use of the corresponding emotion regulation strategy.

The Barratt Impulsivity Scale-Brief (BIS-Brief) was used to assess impulsivity [26]. This scale utilizes 8 items from the Korean version of the BIS-11, which has been evaluated for its validity and reliability [27]. Each item is rated on a 4-point Likert scale. Higher scores indicate higher impulsivity.

The Children’s Revised Impact of Event Scale (CRIES) [28] was used to assess the degree of PTSD symptoms. The CRIES is a 13-item self-report scale adapted from the Impact of Event Scale [29]. Each item is scored on a four-point Likert scale (0 = “not at all”; 3 = “often”). Higher scores indicate higher PTSD symptoms.

#### 2.2.4. Suicidal Ideation

Suicidal ideation was measured with a single question asking participants “Have you ever seriously considered suicide in the last 12 months?” Participants who stated that they had a suicidal ideation in the last 12 months were classified as high risk group and were consulted by a physician if needed.

### 2.3. Statistical Analyses

We compared the sociodemographic characteristics of individuals with and without suicidal ideation using the independent *t*-test for continuous variables and chi-square test for categorical variables. Then, we investigated the difference between familial, social, and psychological characteristics of those with and without suicidal ideation. According to the Kolmogorov–Smirnov normality test, none of these variables had a normal distribution. Therefore, the Mann–Whitney U test was performed. Also, the effect size for each factor was retrieved, and those with medium size effect (Cohen’s *d* > 0.5) were accepted [30]. We then conducted a multiple binary logistic regression analysis with suicidal ideation as the main outcome variable, and the variables that significantly differed between suicidal and non-suicidal participants as the principal predictors. All statistical analyses were performed using SPSS Statistics 21.0 (IBM Corp, Armonk, NY, USA). Statistical significance was defined as an alpha of less than 0.05.

## 3. Results

Table 1 shows the sociodemographic characteristics of the NKR youths with and without suicidal ideation. We observed no significant group differences in terms of sex, age, parental origin, parental educational levels, or type of residence.

Table 2 shows the familial, social, and psychological characteristics of NKR youths with and without suicidal ideation. NKR youths with suicidal ideation reported lower familial cohesion compared to those without suicidal ideation. The former group also had lower resilience and self-esteem, higher PTSD symptoms, and used emotional suppression more frequently compared to the latter group. There was a difference between familial adaptability between NKR youths with and without suicidal ideation, however, the effect was small.

Table 3 shows the results of the multiple regression analysis to identify the independent predictors of suicidal ideation. Only those factors with Cohen’s *d* > 0.5 and *p* < 0.05 from Table 2 were included in the analysis. Emotional suppression and familial cohesion were significantly associated with suicidal ideation in this model.

## 4. Discussion

This study explored the factors from the individual, family, and community level that are related to suicidal ideation of NKR youths. We found that NKR youths with suicidal ideation have lower level of familial cohesion, self-esteem, and resilience, with higher emotional suppression and PTSD symptoms than those without suicidal ideation. Moreover, according to the multiple regression analysis, emotional suppression and familial cohesion were associated with suicidal ideation. This shows that factors from individual and family level are associated with suicidal ideation of NKR youths.

In this study, 16.7% of the NKR youths reported having suicidal ideation in the past 12 months. According to our data, there were no significant differences in sociodemographic variables between youths with and without suicidal ideation. Past review studies [2,8] have found mixed results with regard to the associations between sociodemographic variables (e.g., sex, age, parental educational level, and residence) and the mental health of refugee youths. Obtaining a clear result is difficult, given the need to consider multiple potential confounders when investigating this association [2]. For instance, when investigating the association between age and mental health, confounders such as age at migration, age-related policies for education, age of first exposure to traumatic events, and many other factors must be considered.

As for familial factors, adolescents with suicidal ideation had significantly lower familial cohesion compared to those without suicidal ideation. Along with this result, multiple logistic regression analysis showed that familial cohesion was associated with lower odds of suicidal ideation, after controlling for other variables. The association between family cohesion and suicidal ideation might be explained by how family is a main source of emotional support [31], and the fact that family cohesion acts as a protective factor for mental problems (i.e., depressive symptoms) among NKRs [32]. NKR youths lose social connections by escaping from their country and settling to a new place. Accordingly, enhancing family cohesion might help adolescents endure traumatic experiences as well as adjust to their new environment, which in turn reduces their likelihood of suicide ideation. Interventions to help NKR youths to settle in Korea should include ways to build stronger familial cohesion.

As for the results concerning PTSD symptoms, these are in line with a case-control study [33] showing that NKRs tend to have more severe PTSD symptoms, on average, compared to South Koreans, and PTSD is known to be associated with suicide [11]. This finding highlights the importance of providing interventions aimed at preventing NKRs from developing PTSD symptoms after their exposure to traumatic events.

After arriving in South Korea, NKR youths must face another obstacle: acculturative stress. Acculturative stress manifests as homesickness, a sense of alienation, culture shock, feelings of marginalization, perceived discrimination, gaps in physical health, and problems in adjusting to their new education system [6,7], all of which lower NKRs’ self-efficacy and self-esteem [6,33,34]. Moreover, various studies [6,34,35,36,37] have found that acculturative stress predicts psychiatric problems, including depression, anxiety, and PTSD. However, resilience might work as a buffer against these mental health problems—in other words, individual differences in resilience might help NKRs in dealing with environmental stress [8]. For instance, in one study [6], acculturative stress was related to greater depression and anxiety symptoms, while ego resiliency acted as a mediator in these relationships. Therefore, our findings that lower levels of self-esteem and resilience among NKRs exhibiting suicidal ideation are compatible with those of previous studies. This implies that programs that could improve NKR youths’ self-efficacy and resilience need to be developed.

NKR youths with suicidal ideation exhibited lower emotional suppression compared to that of youths without suicidal ideation. Moreover, in the multiple regression analysis, emotional suppression was associated with higher odds of suicidal ideation of NKR youths. Alexithymia, defined as a difficulty in identifying, describing, and/or expressing emotions, has been suggested as a risk factor of psychiatric problems such as PTSD [38,39]. Indeed, Park, et al. [40] emphasized the important of expressing emotions among NKRs, stating that clearly identifying and expressing emotions alleviated PTSD symptoms among NKRs. Our result indicates that emotional suppression is not only associated with PTSD symptoms, but also suicidal ideation of NKR youths. This implies that helping NKR youths express their emotions after their traumatic experience is important for their mental health.

This study had several limitations. First, this study utilized a cross-sectional design. Therefore, we cannot infer any causal relationships between the studied variables and suicidal ideation among NKR youths. Next, the number of participants in our study was relatively small, with only 29 youths exhibiting suicidal ideation; this might influence the external validity of our results. For example, the small sample size might account for the negative results in the social support factor. Second, participants were students of only two schools, which mean that they were not likely drawn from a representative sample. Third, the data were collected through self-reports, and suicidal ideation was measured with a single question, which might have resulted in reporting bias [41]. Especially since suicidal ideation was measured with a single question, future studies need to use a validated scale to replicate our study. Fourth, we did not explore factors included in the societal level from the conceptual framework [1] we have referred to. Since NKR youths face various obstacles, including acculturative stress [6,7], factors included in the societal level need to be considered in future studies. Finally, the conceptual framework [1] that we have referred to was developed based on Bronfenbrenner’s model [19]. Since we did not use the fundamental model, we may have missed some aspects from the model which could be associated with NKR youth’s suicidal ideation.

Despite the aforementioned limitations, this study had several meaningful results. Specifically, NKR youths are at risk of suicide. To lower this risk, familial and individual interventions, particularly those focusing on emotional expression and familial cohesion, are essential. We believe that our results will help experts in related fields intervene in NKR youths’ suicidal ideation. Further studies using a larger, more representative sample, and a longitudinal design, are needed to confirm our results. Additionally, future studies should aim to develop intervention programs for NKR youths and examine their effectiveness.

## Figures and Tables

**Table 1:** Sociodemographic characteristics of North Korean refugee youths with and without suicidal ideation.

Characteristics	Without Suicidal Ideation (*N* = 145)	With Suicidal Ideation (*N* = 29)	$\chi^{2}/t$	*p*
	*N* (%)	*N* (%)		
Sex, male	46 (31.7)	11 (37.9)	0.42	0.516
Age, mean (SD)	18.86 (2.86)	18.97 (3.41)	0.17	0.864
Birthplace			1.34	0.247
North Korea	82 (56.6)	13 (44.8)		
China	63 (43.4)	16 (55.2)		
Paternal educational level			0.04	0.851
High school degree or lower	97 (74.6)	16 (72.7)		
College degree or higher	33 (25.4)	6 (27.3)		
Maternal educational level			1.68	0.195
High school degree or lower	102 (73.9)	12 (60.0)		
College degree or higher	36 (26.1)	8 (40.0)		
Residence			0.10	0.752
With family	56 (38.9)	10 (35.7)		
With relatives/friends/alone or in a facility	88 (61.1)	18 (64.3)		

**Table 2:** Familial, social, and psychological characteristics of North Korean refugee youths with and without suicidal ideation.

Characteristics	Without Suicidal Ideation (*N* = 145)	With Suicidal Ideation (*N* = 29)	Mann-Whitney U	*p*	Cohen’s *d*
	Mean Rank	Mean Rank			
Familial					
Familial adaptability	91.94	65.31	1459.0	0.009	0.40
Familial cohesion	93.51	57.47	1231.5	<0.001	0.55
Social					
Psychological support	90.28	73.62	1700.0	0.100	0.25
Practical support	90.25	73.76	1704.0	0.105	0.25
Psychological					
Resilience	93.79	56.03	1190.0	<0.001	0.58
Self-esteem	94.88	50.60	1032.0	<0.001	0.69
Cognitive reappraisal	90.29	73.57	1698.5	0.100	0.25
Emotional suppression	81.29	118.53	1202.5	<0.001	0.57
Impulsivity	85.86	95.71	1864.5	0.334	0.15
PTSD symptoms	81.55	114.07	1303.0	0.001	0.51

Abbreviations: PTSD, post-traumatic stress disorder.

**Table 3:** Variables associated with suicidal ideation in North Korean refugee youths.

Characteristics	AOR (95% CI)	*p*
Familial cohesion	0. 94 (0.89–0.99)	0.039
Resilience	0.88 (0.77–1.00)	0.053
Self-esteem	0. 93 (0.84–1.02)	0.111
Emotional suppression	1. 32 (1.08–1.60)	0.006
PTSD symptoms	1.02 (0.99–1.06)	0.183

Abbreviations: PTSD, post-traumatic stress disorder; AOR, adjusted odds ratio.
